# The civil society monitoring of hepatitis C response related to the WHO 2030 elimination goals in 35 European countries

**DOI:** 10.1186/s12954-020-00439-3

**Published:** 2020-11-19

**Authors:** M. Maticic, Z. Pirnat, A. Leicht, R. Zimmermann, T. Windelinck, M. Jauffret-Roustide, E. Duffell, T. Tammi, E. Schatz

**Affiliations:** 1 Faculty of Medicine, University of Ljubljana, Ljubljana, Slovenia; 2 Clinic for Infectious Diseases and Febrile Illnesses, University Medical Centre Ljubljana, Ljubljana, Slovenia; 3 Fixpunkt E. V., Berlin, Germany; 4 Department of Infectious Disease Epidemiology, Robert Koch Institute, Berlin, Germany; 5 Free Clinic, Antwerp, Belgium; 6 Cermes3 (Inserm U988/CNRS 8211/EHESS/Université de Paris), Paris, France; 7 European Centre for Disease Prevention and Control, Stockholm, Sweden; 8 THL, Helsinki, Finland; 9 Correlation-European Harm Reduction Network, Amsterdam, The Netherlands

**Keywords:** Hepatitis C, People who inject drugs, Continuum of care, Harm reduction, Civil society, Monitoring

## Abstract

**Background:**

People who inject drugs (PWID) account for the majority of new cases of hepatitis C virus (HCV) infection in Europe; however, HCV testing, and treatment for PWID remain suboptimal. With the advent of direct acting antivirals (DAAs) the World Health Organization (WHO) adopted a strategy to eliminate HCV as public health threat by 2030. To achieve this, key policies for PWID must be implemented and HCV continuum of care needs to be monitored. This study presents results of the first monitoring led by civil society that provide harm reduction services for PWID.

**Methods:**

In 2019, harm reduction civil society organizations representing focal points of Correlation-European Harm Reduction Network in 36 European countries were invited to complete a 27-item online survey on four strategic fields: use/impact of guidelines on HCV testing and treatment for PWID, availability/functioning of continuum of care, changes compared to the previous year and, the role of harm reduction services and non-governmental organizations (NGOs) of PWID. A descriptive analysis of the responses was undertaken.

**Results:**

The response rate was 97.2%. Six countries reported having no guidelines on HCV treatment (17.1%). Twenty-three (65.7%) reported having treatment guidelines with specific measures for PWID; guidelines that impact on accessibility to HCV testing/treatment and improve access to harm reduction services in 95.6% and 86.3% of them, respectively. DAAs were available in 97.1% of countries; in 26.4% of them they were contraindicated for active drug users. HCV screening/confirmatory tests performed at harm reduction services/community centers, prisons and drug dependence clinics were reported from 80.0%/25.7%, 60.0%/48.6%, and 62.9%/34.3% of countries, respectively. Provision of DAAs at drug dependence clinics and prisons was reported from 34.3 to 42.9% of countries, respectively. Compared to the previous year, HCV awareness campaigns, testing and treatment on service providers’ own locations were reported to increase in 42.9%, 51.4% and 42.9% of countries, respectively. NGOs of PWID conducted awareness campaigns on HCV interventions in 68.9% of countries, and 25.7% of countries had no such support.

**Conclusion:**

Further improvements in continuum-of-care interventions for PWID are needed, which could be achieved by including harm reduction and PWID organizations in strategic planning of testing and treatment and in efforts to monitor progress toward WHO 2030 elimination goal.

## Introduction

People who inject drugs (PWID) account for the majority of new cases of hepatitis C virus (HCV) infection in high income countries [1]. Globally, 8.5% of the estimated 71 million HCV infections occur among persons aged 15–64 years who injected drugs within the last 12 months [1]. In the WHO European region, an estimated two million PWID are living with active HCV infection, about 75% of whom are thought to live in Eastern European countries. In available national studies, the prevalence of HCV antibodies among PWID varies widely from 15% in the Czech Republic to 82% in Portugal, reflecting the real differences among the populations of PWID in different countries [2, 3]. It was estimated that in 2015, 16% of all people living with acute or chronic HCV infection in the European Union (EU) and Norway were PWID [4, 5]. Among the four countries collecting data on the prevalence of viraemic HCV infections in PWID including acute and chronic ones, the prevalence in 2017 ranged from 26.7% in England and Wales to 65.1% in Vienna [6].

Chronic HCV infection causes liver damage that may proceed to cirrhosis, end-stage liver disease, and hepatocellular carcinoma [7]. Several studies have shown that treatment with direct acting antivirals (DAAs) in PWID is as effective as in the general population. Evidence already exists that aside of harm reduction programs, unrestricted and immediately accessible DAAs can lower the HCV prevalence among PWID [8, 9]*.* However, in the last decade, due to aging of HCV chronically infected PWID with untreated HCV infection and late presentation, the mortality from HCV infection has increased particularly in this marginalized group, and deaths from liver disease are now as common as deaths from overdose in PWID over 50 years of age [10, 11].

In 2016, the World Health Organization (WHO) adopted a strategy to eliminate hepatitis C as a public health threat with targets aiming for a 65% decrease in mortality from HCV infection and a 90% decrease of new chronic HCV infections by the year 2030 [11]. To achieve this goal, the countries need to implement key policies and set up an appropriate healthcare system, particularly taking account of the needs of PWID. However, HCV testing and treatment for PWID remain suboptimal. A majority of them lacks access to harm reduction services, in spite of evidence-based recommendations from WHO, the European Association for the Study of the Liver (EASL) [12] as well as other professional associations to assure PWID’s access to HCV testing and care as a matter of priority for individual as well as public health. The availability of hepatitis care varies substantially among countries and often remains below WHO targets, with globally less than 1% of PWID living in countries with access to both, HCV testing and treatment [13–15]. Moreover, even where the services exist, PWID face many difficulties in accessing a continuum of care for hepatitis C that includes prevention, testing, linkage-to-care, and treatment and are often excluded from treatment by restrictive guidelines, have poor access to health services, and are likely to experience stigmatization when discussing/disclosing drug use practices [15].

To follow up the current situation and document progress made toward the 2030 WHO goal, the key policies, particularly for PWID, and a continuum of care should be carefully monitored. A continuum of care represents synergistic interventions for prevention, testing, linkage-to-care, treatment and chronic care which are at the core of an effective hepatitis C response [12, 16]. WHO Europe and the European Centre for Disease Prevention and Control (ECDC) have been working closely with experts in European countries on the monitoring system to help countries assess progress toward eliminating hepatitis C [17], and the European Monitoring Centre for Drugs and Drug Addiction (EMCDDA) has developed an “elimination barometer”, which brings together available data on 17 PWID-specific indicators, matching the WHO’s monitoring and evaluation framework [4]. However, the main problem of reports coming from the monitoring systems is the lack of appropriate data.

To better understand the barriers and opportunities to HCV testing and treatment in PWID, a much greater involvement of first-line service providers such as the harm reduction agencies as well as the drug user community in the development of HCV policy and practice is needed [18]. In order to contribute to the European monitoring efforts from a civil society perspective, the Correlation-European Harm Reduction Network (C-EHRN) decided to develop a framework for a civil society-led monitoring. The C-EHRN is a European civil society network of organizations and individuals with grassroot expertise in the field of drug use, harm reduction, and social inclusion, taking into account also the impact of HCV infection on the well-being of PWID. It is hosted by the Regenboog Groep in Amsterdam, the Netherlands, and co-funded by the European Union [19]. Together with a wide range of partners from across Europe, the C-EHRN developed and implemented a substantial number of surveys, tools, training and advocacy materials aimed at supporting the integration of HCV-related activities as a regular practice within the field of harm reduction service provision [20]. In 2018, C-EHRN collected the experiences of civil society organizations (CSO) providing harm reduction services on interventions in the HCV continuum of care and best practice examples [21, 22]. Furthermore, C-EHRN also conducted a telephone survey on the legal barriers for providing HCV community testing in Europe [23].

In 2019, C-EHRN introduced a novel and complementary monitoring tool in support of European level monitoring of progress toward the WHO elimination goals. This tool aims to collect the experiences of CSO that provide harm reduction services on the availability and access of interventions that constitute the HCV continuum of care. The analysis of the results of this first monitoring is presented in this article.

## Materials and methods

In 2019, a cross-sectional prospective survey was performed by the C-EHRN. It was prepared, conducted and analyzed through multiple rounds of consultation with and input from the members of a multidisciplinary Hepatitis C study group of the C-EHRN, which included the C-EHRN board and an international team of advisers, composed of clinicians, epidemiologists, sociologists, public health specialists, CSO managers and others.

### Data collection scope

Respondents invited for the monitoring were harm reduction CSOs coming from 36 European countries where C-EHRN has its focal points. Scotland was treated separately from the rest of the UK due to the autonomous system for HCV management; the UK data therefore excluded the data from Scotland.

Compared to EMCDDA reports [2, 4], the C-EHRN network brings information from additional nine countries which are not members of the EU (Albania, Bosnia and Hercegovina, Georgia, Montenegro, Russia, Serbia, Switzerland, Ukraine, and North Macedonia); however, no contributions were available from four EMCDDA reporting countries (Cyprus, Estonia, Malta, Turkey).

The “Focal Points” are C-EHRN organizational members and in particular need to fulfil certain criteria such as their willingness to commit to the network’s principles, mission and vision on national and European level, proven thematic expertise in the field of drug use and harm reduction, connectedness on national and European level, and the ability to fulfil the role of intermediary on national level [19]. The selection of focal points for the purpose of monitoring was based on the C-EHRN member assessment in the beginning of 2018. They were selected due to their expected capability to capture the national situation and their proven track record on harm reduction policy and practice.

Participants were invited to join the survey by completing an online questionnaire distributed to respondents via email and/or online. One questionnaire was completed per country. Data was collected by C-EHRN between June and September 2019. After the data collection was achieved, the responses were reviewed and analyzed by the Hepatitis C study group of C-EHRN. In case of unclear, incomplete or inconsistent responses the respondents were asked via email to recheck them. If repeatedly giving unclear information respondents were contacted by phone to obtain a clarification and/or validate the meaning of their response.

### The questionnaire

A 27-item online questionnaire was designed for the purpose of this survey (available at: https://www.correlation-net.org/wp-content/uploads/2020/01/C-EHRN_Monitoring_tool.pdf). It was based on the experiences of previous work of the C-EHRN and the input of external experts, mainly practitioners from the field [24]. Based on the C-EHRN member assessment at the beginning of 2018, thematic expert groups were established, consisting of six experts, invited by the C-EHRN office, who contributed on a voluntary base to the different activities. The HCV expert group was asked to review the proposed structure and focus of the monitoring questions and to comment on criteria and indicators and to contribute to the analysis of data and/or the monitoring report finally. This process was supported by a Scientific Expert Group (SEG), which includes researchers from organizations within and outside of C-EHRN, including the EMCDDA. The SEG developed, adapted and reviewed the monitoring activities in close cooperation with the thematic expert group on HCV.

The questionnaire addressed four strategic fields: the use and impact of guidelines on the accessibility to HCV testing and treatment for PWID; the availability and functioning of a continuum of care in different countries and regions; changes in continuum-of-care services compared to the previous year; and, the role of harm reduction services and non-governmental organizations (NGOs) of PWID in this context.

The definition of PWID used in this study included three different groups: “active PWID” referred to those who had injected drugs within the past 6 months [14]; “PWID on Opioid Substitution Therapy (OST)” referred to those who are currently included in an OST program and are either not injecting anymore or are still occasionally injecting drugs; and “former PWID” referred to those who completely stopped injecting drugs.

The answers to most of the questions were binary (“yes”/“no”); however, some questions had multiple-choice answer options. Furthermore, a free-text box was offered which respondents could use to add comments to clarify their answers and to provide additional qualitative information, links and other sources. The questionnaire was administered in English since no language barriers were expected from C-EHRN focal points respondents.

### Data analysis

A descriptive and geospatial analysis was performed by the Hepatitis Study group of the C-EHRN. For every question and all the respondents the counts summaries and frequencies were performed. The comments in the boxes were analyzed separately, and in case of several similar comments or description of unusual practices or particularities, these are described in the paper.

## Results

Out of 36 invited C-EHRN focal points, all except one (from Estonia) responded (35/36, 97.2%).

### The use of guidelines for hepatitis C treatment in people who inject drugs

**Fig. 1:**
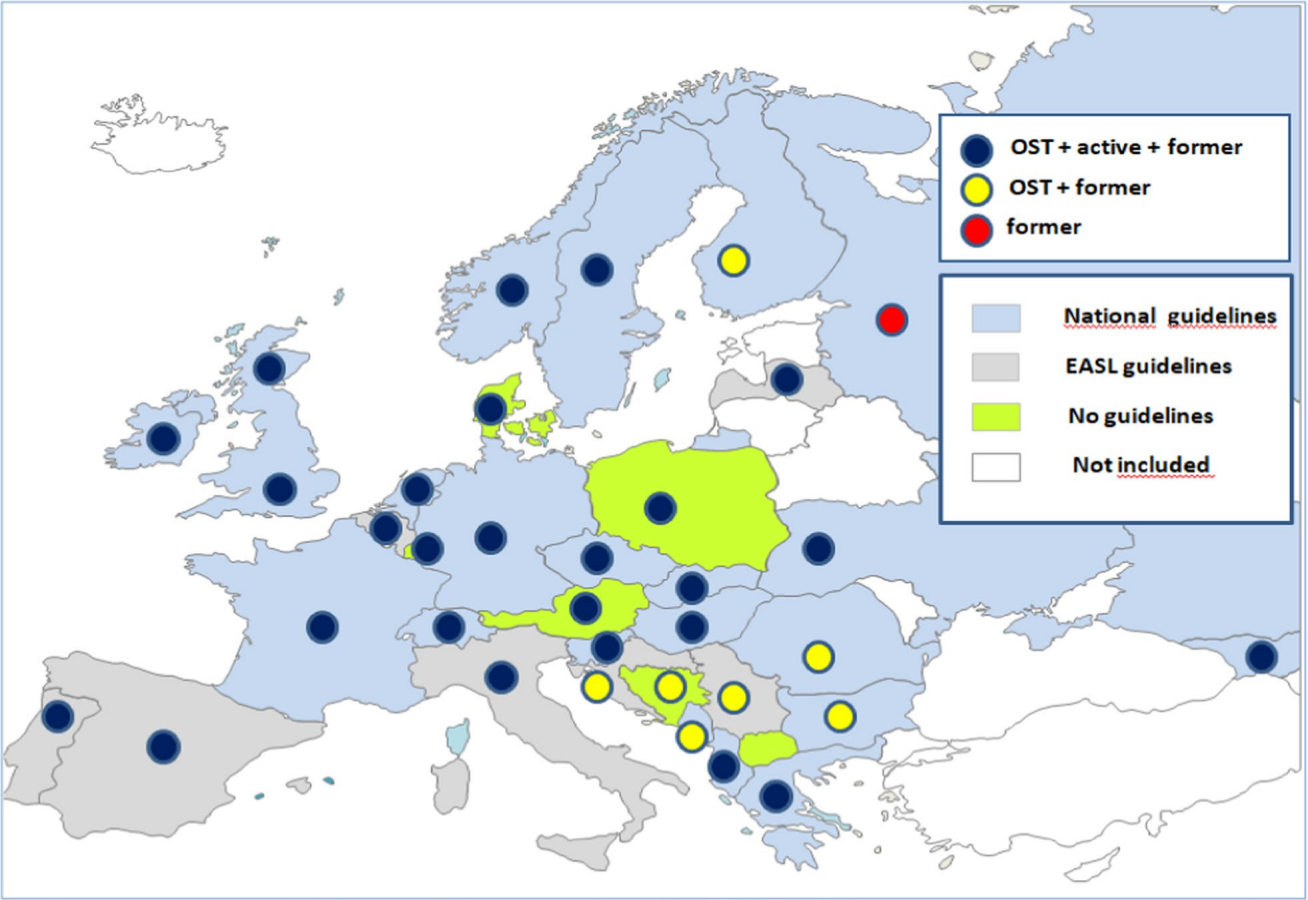
Reported use of most relevant guidelines for the treatment of hepatitis C from 35 European countries and their indications for treatment in different groups of people who inject drugs. ^#^Scotland was treated separately from the rest of the UK. ^##^The countries in white did not participate in the study. ^###^In North Macedonia treatment for hepatitis C is not available at all

Among respondents, six (17.1%) reported on still having no national guidelines for HCV treatment and seven (20.0%) reported on using the ones from EASL (Fig. 1).

In only 23 of the responding countries (65.7%), the guidelines used include specific measures for PWID. In all but one of those 23 countries the guidelines somehow impact the accessibility to HCV testing and treatment of PWID; however, they impact better access to harm reduction service in only 19/22 countries (Table 1). Qualitative responses showed that several respondents were pessimistic about the impact of the guidelines used in their country on better access of PWID to the services such as testing and treatment and even by their own agencies. Responses received indicated that even if national guidelines exist, they have a limited relevance in practice. A range of challenges was reported, such as outdated guidelines and complicated testing and treatment systems, as well as lack of services and other kinds of disproportions between the formal guidelines and the real-life situation. Criminalization of a possession and use of drugs for personal reasons was also reported as a barrier leading to discrepancy between the policy and real-life guidelines. However, as stated by some of the respondents, harm reduction agencies did not necessarily need official guidelines to start interventions on HCV.

**Table 1:** Specific measures for hepatitis C treatment in people who inject drugs and barriers observed in harm reduction services, as reported from 35 European countries

Country	Specific for PWID						Barriers in HR services		
	Linkage-to-care protocol	Treatment guidelines	Guidelines impact access to testing/treatment	Guidelines impact access to HR services	Discrepancy in DAA use between policy and practice	Automatic reimbursement of DAA	Lack of funding/political support/general recognition of HR	Shortage of know-ledge/training/skilful staff	Law
Albania	Y	Y	Y	Y	Y	Y	Y	na	na
Austria	N	N/A	N/A	N/A	N	Y	na	na	na
Belgium	N	Y	Y	Y	N	Y	na	na	na
Bosnia and Hercegovina	na	N/A	N/A	N/A	N	Y	na	na	na
Bulgaria	N	N	Y	N	Y	Y	na	na	na
Croatia	Y	Y	Y	N	N	Y	na	na	na
CzechRepublic	N	Y	Y	Y	N	Y	na	Y	na
Denmark	na	N/A	N/A	N/A	N	Y	na	na	na
Finland	N	Y	Y	Y	Y	Y	na	na	na
France	Y	Y	Y	Y	N	Y	Y	Y	na
Georgia	Y	Y	Y	Y	N	Y	na	na	na
Germany	Y	Y	Y	Y	N	Y	Y	Y	na
Greece	Y	Y	Y	Y	N	Y	na	na	Y
Hungary	N	Y	Y	N	Y	N	Y	na	na
Ireland	N	Y	N	Y	N	Y	Y	Y	na
Italy	N	Y	N	N	N	Y	na	na	na
Latvia	Y	Y	MI	MI	N	Y	na	na	na
Luxembourg	Y	N/A	N/A	N/A	N	Y	na	na	na
Montenegro	Y	Y	Y	N	N	Y	na	na	Y
Macedonia, North	N	N/A	N/A	N/A	N/A	Y	Y	na	na
Netherlands	Y	N	Y	N	N	Y	na	na	na
Norway	Y	Y	Y	Y	N	Y	na	na	na
Poland	N	N/A	N/A	N/A	Y	Y	na	na	na
Portugal	Y	Y	Y	Y	N	Y	na	na	na
Romania	na	N	Y	Y	Y	N	Y	na	na
Russia	N	N	N	N	Y	Y	na	Y	na
Scotland	Y	Y	Y	N	N	Y	na	na	na
Serbia	Y	Y	N	N	Y	N	Y	na	na
Slovakia	N	N	N	N	Y	Y	na	na	na
Slovenia	Y	Y	Y	Y	N	Y	na	na	na
Spain	Y	Y	Y	Y	N	Y	na	na	na
Sweden	Y	Y	Y	Y	N	Y	na	na	na
Switzerland	N	Y	Y	Y	N	Y	na	na	na
Ukraine	Y	Y	Y	Y	Y	Y	na	na	na
UK	N	N	N	Y	N	N	Y	na	na

Scotland was treated separately from the rest of the UK

*N* no, *Y* yes, *na* not analyzed, *N/A* not applicable because there are no national guidelines, *DAA* direct acting antivirals, *HR* harm reduction

According to the respondents, the DAAs were available in all reporting countries but North Macedonia (34/35, 97.1%). However, from 11/34 countries (32.4%) an official policy on restrictions for the use of DAAs was reported (Albania, Croatia, Finland, Latvia, Luxembourg, Montenegro, Romania, Russia, Serbia, Sweden, Ukraine). In 10 out of 34 countries (29.4%) DAAs were reported to be accessible only for people presenting liver fibrosis; in two countries (2/34, 5.9%) only advanced fibrosis or cirrhosis represented indications for DAA treatment (Albania, Serbia).

In 9/34 countries (26.4%) active drug users were still not applicable for DAA treatment (Fig. 1). With the exception of Russia, PWID on OST were allowed to get HCV treatment in all other countries (33/34, 97.1%); former injectors were allowed DAA treatment in all of the included countries where DAAs were available (34/34, 100%).

All but six respondents (28/34, 82.3%) assessed that DAAs were being used in practice as stated in the official policy documents (Table 1). DAA treatment was reimbursed by the health insurance or public health services in all countries except the UK; however, in a few countries, the treatment was not automatically reimbursed for PWID (Table 1).

### The functioning of a continuum of care for people who inject drugs

The C-EHRN monitoring data on a continuum of care including HCV testing and treatment showed that within Europe, a variety of service options existed for PWID, with some good practice examples as well as some bad ones, providing testing and treatment in a very limited variety of settings (Table 2).

**Table 2:** Settings for hepatitis C testing and treatment, as reported from 35 European countries

Country	Gastro-enterology clinics		Infectious disease clinics		Drug dependence clinics		Harm reduction services or community centers		General practitioner		Pharmacy		Prison	
	Test	Treat	Test	Treat	Test	Treat	Test	Treat	Test	Treat	Test	Treat	Test	Treat
Albania	A R	Y	A R	Y	A	N	A	na	N	N	N	N	A	N
Austria	A R	Y	N	N	A R	N	A	na	A R	N	N	N	A R	N
Belgium	A R	Y	A R	N	A	N	A	na	A	N	N	N	A	Y
Bosnia and Hercegovina	N	Y	A R	N	A	N	A	na	N	N	N	N	A	N
Bulgaria	R	Y	N	N	N	N	A R	na	N	N	N	N	N	N
Croatia	A R	Y	A R	Y	A	N	A	na	A R	N	N	N	(A)	N
Czech Republic	A R	Y	A R	Y	A	Y	A	na	R	Y	N	N	A R	Y
Denmark	N	N	A R	Y	A R	N	A	na	A R	N	N	N	N	N
Finland	R	Y	A R	Y	A R	Y	A R	na	A R	Y	N	N	A R	Y
France	A R	Y	A R	Y	A	Y	A	na	A R	Y	N	N	A R	Y
Georgia	A	Y	A R	Y	A	Y	A R	na	A	Y	N	N	A	Y
Germany	R	Y	A R	Y	N	Y	A R	na	A R	Y	N	N	A R	Y
Greece	N	N	R	N	N	N	A	na	N	N	N	Y	N	N
Hungary	N	Y	R	Y	N	N	A	na	N	N	N	N	A R	N
Ireland	R	Y	R	Y	R	Y	N	na	R	N	N	N	R	Y
Italy	N	N	R	Y	A	N	A	na	N	N	A	N	A R	Y
Latvia	N	N	R	Y	N	N	A	na	R	N	N	N	R	Y
Luxembourg	N	N	A R	Y	A	N	N	na	A	N	N	N	A	N
Montenegro	N	N	R	Y	N	N	N	na	N	N	N	N	N	N
Macedonia, North	N	Y	R	Y	N	N	N	na	N	N	N	N	N	N
Netherlands	A R	Y	A R	Y	A	N	N	na	A R	N	N	N	A R	N
Norway	A	Y	N	N	N	N	N	na	A R	N	N	N	N	N
Poland	N	N	R	Y	A	N	A	na	A	N	N	N	A	Y
Portugal	A R	Y	A R	Y	A R	Y	A R	na	R	N	N	N	A R	Y
Romania	R	Y	R	Y	A	N	A	na	A	N	N	N	A	N
Russia	N	N	A R	Y	A	N	A	na	N	N	N	N	N	N
Scotland	A R	Y	A R	Y	A R	Y	A R	na	A R	Y	A R	Y	A R	Y
Serbia	N	N	N	Y	N	N	A	na	N	N	N	N	N	N
Slovakia	R	Y	N	Y	R	N	A	na	N	N	N	N	R	N
Slovenia	N	Y	R	Y	R	N	R	na	R	N	N	N	R	N
Spain	R	Y	R	Y	A R	Y	A R	na	R	N	N	N	R	Y
Sweden	A	N	A R	Y	A R	Y	A R	na	A	N	N	N	A R	Y
Switzerland	A R	Y	A R	Y	A R	Y	A	na	A R	N	N	N	N	N
Ukraine	N	N	A R	Y	N	N	A	na	A	N	N	N	A	N
UK	A R	Y	A R	Y	A R	Y	A R	na	A	N	A	N	A R	Y

Scotland was treated separately from the rest of the UK

*A* antibody test, *R* RNA test, *N* no, *Y* yes, *na* not analyzed

^#^Testing included either screening test for hepatitis C virus antibodies (A) or confirmatory test for hepatitis C virus RNA (R), or both (AR)

^##^Treatment in harm reduction services and community centers was not included in the questionnaire

Respondents reported that screening tests for the detection of anti-HCV antibodies included either saliva testing (oral swabs) or blood testing (finger prick), whereas detection of HCV RNA was used as a confirmatory test. In the majority of countries the screening tests were a standard of care also outside the medical settings, such as harm reduction services or community centers (28/35, 80.0%) and prisons (21/35, 60.0%), as well as drug dependence clinics (22/35, 62.9%) (Table 2). The confirmatory testing was much more commonly performed at the infectious disease clinics (30/35, 85.7%) and gastroenterology clinics (18/35, 51.4%) compared to other settings, such as drug dependence clinics (12/35, 34.3%) and harm reduction services (9/35, 25.7%); however it was performed in prisons in 17/35 (48.6%) countries (Table 2).

The prioritized settings for DAA treatment were the two clinical settings, infectious diseases and gastroenterology (29/35, 82.9% and 24/35, 68.6%, respectively). General practitioners (GPs) performed screening and confirmatory testing in 18/35 (51.4%) and 16/35 (45.7%) countries, respectively whereas they were allowed to prescribe DAA treatment in only 6/35 (17.1%) countries (the Czech Republic, Finland, France, Georgia, Germany, Scotland). DAA treatment was provided at the drug dependence clinics in 12/35 (34.3%) countries, and it was also provided in prisons in 15/35 (42.9%) countries (Table 2). Since May 2019, all the physicians in France were allowed to prescribe DAAs.

Pharmacies were very rarely used as a setting for HCV testing (Italy, Scotland, and the UK) and DAA treatment (Scotland) (Table 2).

Scotland was reported to be the only country that offered HCV screening and confirmatory testing as well as DAA treatment at all the settings mentioned above.

Eighteen countries (18/35, 51.4%) reported having precise linkage-to-care protocols/guidelines for newly HCV diagnosed PWID to be referred for treatment (Table 1). The government monitored the numbers/proportions of people who progress through each stage of the HCV continuum of care on the national level in 14/35 countries (40.0%); monitoring at the regional or local level was performed in five and seven countries, respectively, whereas in the remaining countries, monitoring was not performed et al.

### Longitudinal evaluation of a continuum of care

**Fig. 2:**
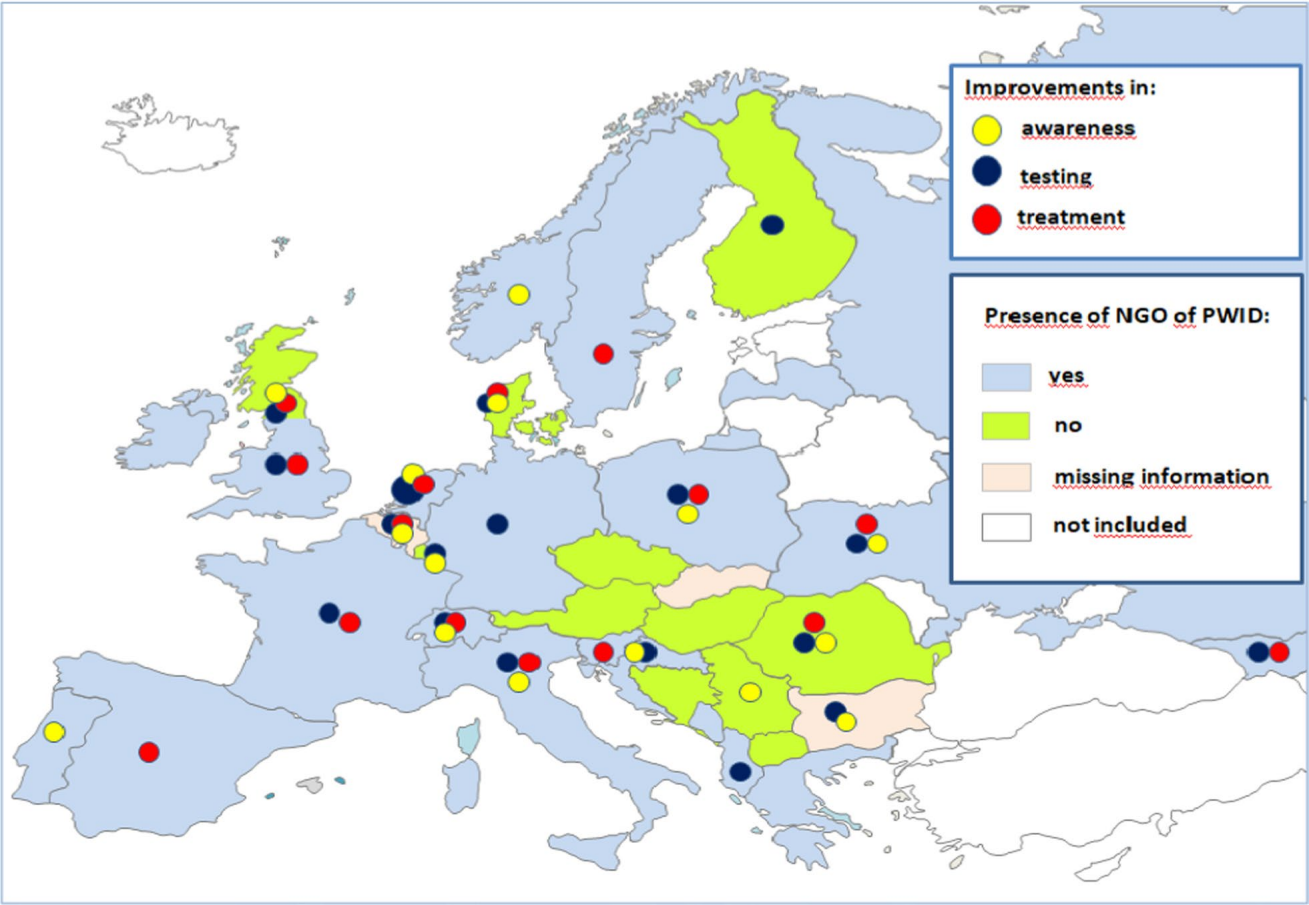
Improvements in a continuum of care compared to the previous year and the role of harm reduction and non-governmental organizations of people who inject drugs, reported from 35 European countries. *NGO* non-governmental organization, *PWID* people who inject drugs. ^#^Scotland was treated separately from the rest of the UK. ^##^The countries in white did not participate in the study

The current C-EHRN survey revealed the dynamic of providers’, investment in various services of a continuum of care. Compared to the previous year, 15/35 countries (42.9%) reported on having more attention paid to HCV awareness campaigns, 18/35 (51.4%) to testing on the service providers’ own locations, and 15/35 (42.9%) to treatment on the service providers’ own locations; 9/35 (25.7%) countries reported on improvements made in all the three services (Belgium, Denmark, Italy, the Netherlands, Poland, Romania, Scotland, Switzerland, Ukraine) (Fig. 2). In other countries, the situation had remained the same as in the previous year or there had been even less activities but overall compared to the previous year the results on changes made in the continuum of care were positive.

### Role of harm reduction and non-governmental organizations of people who inject drugs

Twenty-four European countries (24/35, 68.6%) reported on having NGOs of PWID that are working actively for political awareness in regard of HCV interventions whereas no such NGO support is reported from nine countries (9/35, 25.7%) (Austria, Bosnia and Herzegovina, the Czech Republic, Finland, Hungary, Luxembourg, Romania, Scotland, Serbia) (Fig. 2). Finally, while trying to address HCV among PWID, the barriers and limitations repeatedly mentioned by the harm reduction organizations were the lack of funding, political support and general recognition of harm reduction measures (Table 1). The shortage of knowledge and training on HCV infection, as well as a lack of skilful staff were mentioned by five countries (Table 1). Another reported barrier was the weakness of the CSO, whereas legal barriers, particularly those regarding the possibility of testing within the community were reported from Greece and Montenegro.

## Discussion

To date, globally, there exists no uniquely standardized protocol or system to monitor and evaluate the progress made toward the elimination of hepatitis C as a public health threat as set out in 2016 by the WHO Global Health Sector Strategy on Hepatitis that includes also a CSO perspective. As known from the global HIV/aids response, CSOs may contribute an exceptional role in fulfilling both formal and informal monitoring functions because their participation creates more inclusive global health governance and contributes to strengthening commitments to human rights [25].

In Europe, the first report of the ECDC monitoring the progress toward HCV elimination in 2019 by collecting data from a range of existing sources in 31 countries of EU/European Economic Area (EEA) highlighted significant gaps in the availability of data related to the continuum of care such as prevention, testing, and treatment. The report showed that overall, 27 countries provided data for at least one of the key stages of the HCV continuum of care, whereas only 11 countries were able to provide data along the continuum [17]. The conclusion was that countries in the EU/EEA were not on track for meeting the WHO 2030 elimination targets.

From another perspective, the 2018 study of the European Liver Patient Association (ELPA) including patient groups from 25 European countries focused on the qualitative implementation of WHO recommendations and verification of policies to eliminate viral hepatitis in each of the examined countries [26]. The results of the study revealed that generally the European region was not on track to meet WHO 2030 HCV goals, and presented some concerning discrepancies among the studied countries as well as overlooked opportunities for high-risk populations in many settings.

For the high-risk population of PWID, in 2019 the EMCDDA established an elimination barometer for hepatitis C helping EU countries, Norway and Turkey to assess their progress toward eliminating HCV among PWID [4]. The current results revealed a high burden of HCV among PWID with information gaps in several countries, absence of systematic collection of data on HCV continuum of care for PWID, missed opportunities to HCV diagnosing and various restrictions to treatment with DAAs.

C-EHRN monitoring data presented here constitute the first results of civil society-led monitoring, reflecting the perspective of harm reduction service providers in 35 European countries evaluating the 2019 HCV treatment guideline situation and progress made between the years 2018 and 2019 in HCV interventions for PWID. It gathered responses on three key stages of a HCV continuum of care in PWID.

The data obtained revealed discrepancies among European countries regarding the HCV treatment guidelines, since some reported on having the national guidelines, others reported on using the EASL ones, whereas in some cases both were in place. Besides, a lack of specific guidelines for HCV treatment in PWID was reported from several countries. There were also big differences within Europe as to where and how PWID could access testing for HCV. Even though incarcerated persons represent a high-risk population for HCV infection [27], HCV testing in prisons was reported only from 21 countries, representing a missed opportunity to identify cases. In 2019 DAAs were available in all countries of the region except North Macedonia; however, PWID were still not allowed access to HCV treatment in 10 European countries. These results reveal a persistent stigmatization toward PWID within the medical system which impedes good access to HCV care for PWID at an individual level, but also favors transmission of HCV infection in the population. Although 23 countries reported having guidelines that included specific HCV management recommendations for PWID, many C-EHRN respondents were somewhat pessimistic about the impact of such guidelines on improving access to the HCV continuum of care in their country, especially to integrated test-and-treat services. There were qualitative explanations for such a gap between the official guidelines and their implementation coming from CSO of several countries. They clearly reveal this well recognized challenge that needs to be addressed as soon as possible. However, once access to DAA treatment had been achieved, the costs were reimbursable by health insurance or through the public health service in all but one monitored country.

On a more positive note, 23 European countries reported PWID organizations working actively to increase political awareness concerning HCV interventions. Compared to 2018, more attention had been paid over the past year to HCV awareness campaigns, to testing at the service providers’ own premises, and to treatment at the service providers own site. However, several barriers to address HCV among PWID are reported to persist, such as a lack of funding, knowledge, recognition, political support and skilful staff as well as weakness of CSO and legal barriers.

The survey also revealed that the monitoring of people progressing through each stage of the HCV continuum of care was performed at the governmental level in less than half of the observed countries, one-third of them reported existence of regional or local monitoring, whereas monitoring of any kind was not at all the practice in one quarter of the included countries.

The analysis of the C-EHRN monitoring for 2019 showed that PWID in particular were still in an unequal position regarding HCV testing and treatment in different European countries and often deprived of proper HCV interventions. When comparing the continuum-of-care situation, it becomes obvious that the integration of testing and treatment at one site is still too rarely the case. However, combination of integrated interventions, such as needle and syringe programs (NSP), OST, access to heroin-assisted treatment and community-based and peer-led harm reduction programs are not only cost effective regarding HCV prevention, but also ensure that marginalized populations stay adhered to services [28]. In countries with progressive HCV treatment policies, such as Scotland, NGOs of PWID have played a pivotal role in raising the issue with the public and advocating for the right of PWID to low threshold HCV testing and treatment [13]. The governments that engage with CSO have been shown to be more advanced in their hepatitis response as demonstrated in the WHO Viral Hepatitis Country Profiles [29].

Indeed, the overall reporting on progress between 2018 and 2019 can be considered positive as there has been more action taking place in several countries. However, to reduce the HCV-related disease burden among PWID and achieve the 2030 elimination goals in Europe, a radical change in the HCV response is still needed in many of the European countries monitored in this C-EHRN survey. National treatment guidelines that address the specific challenges to overcome barriers like stigmatization and criminalization of PWID are still needed in Europe. Those recommendations should underline the necessity of unrestricted access to DAA treatment, improvements in the continuum of care and further development of single site testing and treatment services [30].

To improve the low uptake of HCV testing and treatment among PWID, it is crucial to include harm reduction and drug user organizations in the continuum of services providing HCV management within every European country [31]. The baseline principles of harm reduction which include trust, non-judgmental attitudes, flexibility to adapt to the needs of clients and the active participation of the community of PWID have enabled harm reduction services to be highly effective in engaging PWID in care and treatment and even offer them HCV treatment on spot [28]. In order to reduce hepatitis C incidence and prevalence among PWID, access to interventions such as low-threshold NSP, as well as OST are essential[11, 32]. OST has proven to be effective for the prevention of HCV infection and combination of OST and high-coverage NSP can reduce HCV incidence by more than 70%. The evaluation framework for the WHO elimination strategy provides clear targets to countries regarding the scale of provision of these measures [11]. The 2019 EMCDDA monitoring data show that only a small proportion of countries have achieved the 2020 target for coverage of NSP but the majority of countries with data have reached the 40% coverage target for OST [2]. At the same time, according to a C-EHRN study from 2018 the level of readiness in harm reduction and community-based organizations in Europe to provide testing and treatment for PWID remains high, yet funding, the attitude of health services toward PWID and harm reduction services in general as well as legal and regulatory practices in many countries have a negative impact on PWID’s access to social and health support [22]. So, C-EHRN developed certain strategic priorities and activities to prevent inequities in access to HCV services, discrimination and stigma toward PWID and harm reduction in general, provide more HCV-related program funding, develop national quality standards for HCV management within community and harm reduction settings, improve data collection of such activities, actively engage PWID in developing HCV strategies, and provide more opportunities for staff training and education.

The most important limitation of this survey is the involvement of only one stakeholder group for information selected from the C-EHRN database of focal point harm reduction CSO. They were not necessarily profoundly familiar with their respective governments’ HCV policies; however, they were excellently familiar with the harm reduction and HCV activities in their local environment and represent therefore “real-life experiences.” The validity of the responses was not cross-referenced with current, official policies, so there exists a possibility of some inaccuracies in respondents’ answers. Given the restrictions in terms of numbers of focal points and their ability to represent a nation as a whole, it has to be emphasized that the primary purpose of this monitoring was not to prepare a representative data collection [33]. Rather, it was to provide a well-grounded critical assessment of the current situation and recent developments in their harm reduction scene, respecting the local, regional or national level of their work.

The next step of a CSO-led monitoring in the framework of C-EHRN was reformulation of the questionnaire to reflect data on policy implementation. Besides, a fine tuning of all sections was made to balance national and local level information as well as qualitative and quantitative data. More questions focus on the local, implementation level, and on the experiences of focal points and their clients. If, on the one hand, the monitoring loses in its ability to reflect a broader European situation focusing on developments at national level, it gains in reflecting fundamental qualitative data on service delivery level that can only be collected by CSOs.

## Conclusions

The results of 2019 C-EHRN civil-society led monitoring of hepatitis C policies and the hepatitis C continuum of care for PWID in 35 European countries show a substantial shortfall and variations and urge for more action. Despite progress reported from several countries, further improvements of the existing continuum-of-care interventions for PWID are needed, which may be achieved by including the harm reduction and drug user organizations in the strategic planning of a continuum of services for HCV testing and treatment. Therefore, the roles and responsibilities not only at every level of the health system, but also beyond it need to be defined with respect to their delivery of hepatitis services. The findings of the C-EHRN Monitoring may provide some important information by first-line service providers to the WHO Global Health Sector Strategy. By involving all stakeholders in the monitoring and reporting of national responses, a significant step forward can be made toward the elimination of HCV as a public health threat by 2030, as set out in the WHO Global Health Sector Strategy on Hepatitis.

## Data Availability

The datasets during and/or analyzed during the current study available from the corresponding author on reasonable request.

## Abbreviations

C-EHRN: Correlation-European Harm Reduction Network
CSO: Civil society organizations
DAAs: Direct-acting antivirals
EASL: European Association for the Study of the Liver
ECDC: European Centre for Disease Prevention and Control
EEA: European economic area
ELPA: European Liver Patient Association
EU: European Union
HCV: Hepatitis C virus
NGO: Non-governmental organization
OST: Opioid substitution therapy
PWID: People who inject drugs
WHO: World Health Organization
